# Pan-European forest maps produced with a combination of earth observation data and national forest inventory plots

**DOI:** 10.1016/j.dib.2025.111613

**Published:** 2025-05-06

**Authors:** Jukka Miettinen, Johannes Breidenbach, Patricia Adame, Radim Adolt, Iciar Alberdi, Oleg Antropov, Ólafur Arnarsson, Rasmus Astrup, Ambros Berger, Jón Bogason, Gherardo Chirici, Piermaria Corona, Giovanni D'Amico, Jiří Fejfar, Christoph Fischer, Florence Gohon, Thomas Gschwantner, Johannes Hertzler, Zsofia Koma, Kari T. Korhonen, Luka Krajnc, Nicolas Latte, Philippe Lejeune, Andrew McCullagh, Marcin Mionskowski, Daniel Moreno-Fernández, Mari Myllymäki, Mats Nilsson, Jérôme Perin, Juho Pitkänen, John Redmond, Thomas Riedel, Johannes Schumacher, Lauri Seitsonen, Laura Sirro, Mitja Skudnik, Arnór Snorrason, Radosław Sroga, Berthold Traub, Björn Traustason, Bertil Westerlund, Stephanie Wurpillot

**Affiliations:** a VTT Technical Research Centre of Finland, VTT, P.O. Box 1000, FI-02044, Finland; b NIBIO Norwegian Institute of Bioeconomy Research, Division of Forest and Forest Resources, Høgskoleveien 8, 1430 Ås, Norway; c Institute of Forest Sciences (ICIFOR, INIA-CSIC), Crta. de A Coruña km 7.5, Madrid, E-28040, Spain; d Forest Management Institute (UHUL), Nábřežní 1326, Brandýs nad Labem, 250 01, Czech Republic; e Land and Forest Iceland, Mógilsá, Reykjavík, 162, Iceland; f Austrian Research Centre for Forests (BFW), Seckendorff-Gudent-Weg 8, Vienna, 1131, Austria; g Dipartimento di Scienze e Tecnologie Agrarie, Università degli Studi di Firenze, Alimentari, Ambientali e Forestali. Via San Bonaventura 13, Firenze, 50145, Italy; h CREA Research Centre for Forestry and Wood, viale Santa Margherita 80, Arezzo, 52100, Italy; i Swiss Federal Research Institute WSL, Zürcherstrasse 111, Birmensdorf, CH 8903, Switzerland; j Institut National de l'information Géographique et Forestière (IGN), Service de l'information Statistique Forestière et Environnementale, Chemin du Château des Barres, Nogent-Sur-Vernisson, 45290, France; k Thünen Institute of Forest Ecosystems, Alfred-Möller-Straße 1, Haus 41/42, Eberswalde, 16225, Germany; l Natural Resources Institute Finland (Luke), P.O. Box 2, Helsinki, FI-00791, Finland; m Slovenian Forestry Institute, Večna pot 2, Ljubljana, 1000, Slovenia; n Gembloux Agro-Bio Tech (GxABT), Forest Resource Management Unit, University of Liège (ULiège), 2 Passage des Déportés, Gembloux, 5030, Belgium; o Forest Service, Department of Agriculture, Food and the Marine, Agriculture House, Kildare Street, Dublin 2, Ireland; p Bureau for Forest Management and Geodesy, Leśników 21, Raszyn, 05-090, Poland; q Department of Forest Resource Management, Swedish University of Agricultural Sciences (SLU), Skogsmarksgränd, Umeå, SE-901 83, Sweden; r Biotechnical Faculty, University of Ljubljana, Jamnikarjeva 101, Ljubljana, 1000, Slovenia

**Keywords:** European forest monitoring system, Remote sensing, In-situ data, Forest attribute maps

## Abstract

The dataset includes Pan-European maps of timber volume (Vol), above-ground biomass (AGB), and deciduous-coniferous proportion (DCP) with a pixel size of 10×10 m for the reference year 2020. In addition, a measure of prediction uncertainty is provided for each pixel. The maps have been created using a combination of a Sentinel-2 mosaic, Copernicus layers, and National Forest Inventory (NFI) data.

The mapping was done with the k-Nearest Neighbour (kNN, k=7) approach with harmonized data of species-specific Vol and AGB from 14 NFIs consisting of approximately 151 000 field plots across Europe. The maps cover 40 European countries, forming a continuous coverage of the western part of the European continent.

A sample of 1/3 of NFI plots was left out for validation, whereas 2/3 of the plots were used for mapping. Maps were created independently for 13 multi-country processing areas. Root-mean-squared-errors (RMSEs) for AGB ranged from 53 % in the Nordic processing area to 73 % in the South-Eastern area. The maps are on average nearly unbiased on European level (1.0 % of the mean AGB), but show significant overestimation for small biomass values (53 % bias for forests with AGB less than 150 t/ha) and underestimation for high biomass values (-55 % bias for forests with AGB higher than 500 t/ha).

The created maps are the first of their kind as they are utilizing a large number of harmonized NFI plot observations and consistent remote sensing data for high-resolution forest attribute mapping. While the published maps can be useful for visualization and other purposes, they are primarily meant as auxiliary information in model-assisted estimation where model-related biases can be mitigated, and field-based estimates improved. Therefore, additional calibration procedures were not applied, and especially high Vol and AGB values tend to be underestimated. We therefore discourage from summarizing map values (pixel counting) over areas in interest, as this may inadvertently result in biased estimates.

Specifications Table

**Table:** 

Subject	Earth & Environmental Sciences
Specific subject area	Pan-European maps of timber volume (Vol), above-ground biomass (AGB), and deciduous-coniferous proportion (DCP) with a pixel size of 10×10 m for year 2020.
Type of data	Uint16 GeoTiff files provided in 500×500 km tiles in the EPSG:3035 - ETRS89-extended / LAEA Europe projection.
Data collection	The maps were produced with the k-Nearest Neighbour (kNN) approach (parameters: k=7, Euclidian distance, weighing inverse to distance). Input data consisted of a harmonized database of species-specific Vol and AGB from 14 National Forest Inventories with around 151 000 field plots across Europe, seven spectral bands of Sentinel-2 satellite (B2, B3, B4, B5, B8, B11, B12) and two auxiliary layers (Copernicus high resolution forest type and tree cover density). The processing was run on the Forestry TEP platform (https://f-tep.com/).
Data source location	The maps cover 40 European countries, forming a continuous coverage of the western part of the European continent.
Data accessibility	Repository name: Zenodo Data identification number: 10.5281/zenodo.13143235 Direct URL to data: 10.5281/zenodo.13143235 Instructions for accessing these data: Download requires no registration or any other procedure
Related research article	None.

## 1 Value of the Data

• The maps allow a visualization of the spatial distribution of key forest attributes across Europe.
• The maps will reduce the uncertainty of estimates when combined with reliable reference data in the model-assisted framework.
• The maps can be the starting point for forecasting models for predicting future forest development that require high-resolution input data.
• The maps provide a base for decision support in regions where no other forest information is available.
• The maps give an impression of the local variation in forest attributes that can be used as pre-information for more in-depth surveys.

## 2 Background

The first European wide growing stock volume and biomass maps combining NFI data with remote sensing datasets were created with 500-1000 m resolution NOAA-AVHRR and MODIS data [1,2]. Since then, several remote sensing based global products covering Europe have been produced using both optical and radar satellite sensors [[3], [4], [5]] reaching as high as 100 m spatial resolution.

Although these types of maps are valuable for many aspects of forest monitoring, relying solely on summarizing remote sensing-based maps over large regions such as entire countries, can lead to significant systematic errors. Therefore, the EU PathFinder project [6] aims to develop and demonstrate a forest monitoring system that allows reporting to European and global policies. A mapping and estimation system integrating the use of remote sensing and field data aims to facilitate effective use of field data with remotely sensed and other auxiliary datasets to produce precise forest information. The maps published here are the first maps produced with the system using a combination of European wide Sentinel-2 imagery and over 150 000 NFI field plots across Europe.

## 3 Data Description

**Fig. 1:**
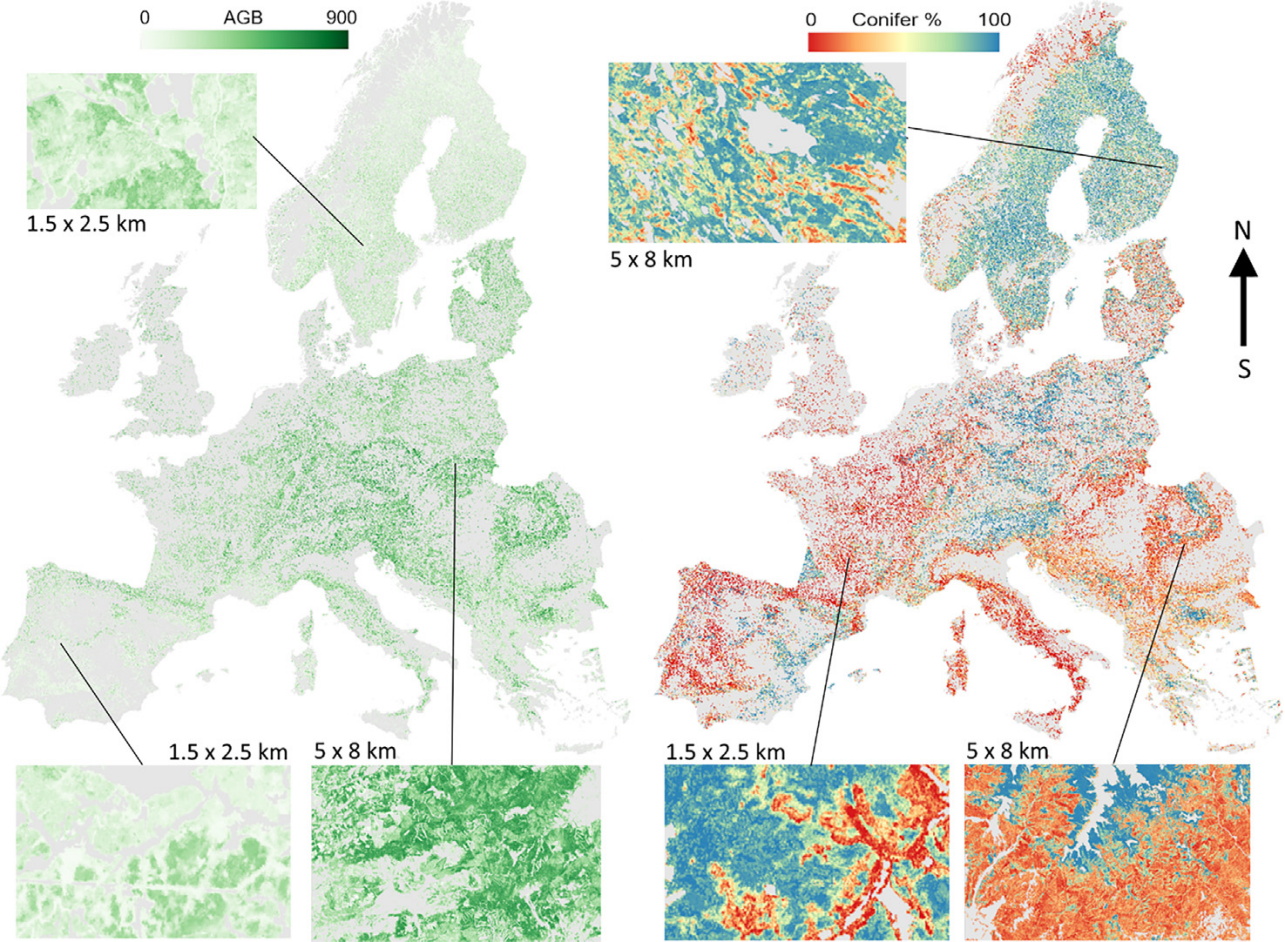
European wide AGB (t/ha) and DCP (%) maps with subsets showing fine details.

Maps of three different target variables are made available, including timber volume (Vol), above-ground biomass (AGB), and deciduous-coniferous proportion (DCP) [7]. While the DCP map provides the percentage of conifers, this allows straightforward calculation of the broadleaf proportion as 100-DCP. Fig. 1 illustrates the AGB map with subsets of AGB and DCP maps from different parts of Europe.

In addition to the target variable maps, also standard deviation layers are provided for each target variable. All of the maps were masked with the Copernicus High Resolution Layer Forest Type (FTY) 2018 forest extent. Areas outside forest have been masked as non-forest and no-data (Table 1).

**Table 1:** Output map technical characteristics.

Variable	Variable naming	Pixel values	Format
**Volume**	Vol (attribute) stdev_vol (uncertainty)	65535: No data 65534: Non-forest Other values: m³/ha	10 m UInt16 GeoTiff
**Above Ground Biomass**	AGB (attribute) stdev_agb (uncertainty)	65535: No data 65534: Non-forest Other values: t/ha	10 m UInt16 GeoTiff
**Conifer proportion**	P_agb_conifers (attribute) stdev_P_agb_conifers (uncertainty)	65535: No data 65534: Non-forest Other values: %	10 m UInt16 GeoTiff

**Fig. 2:**
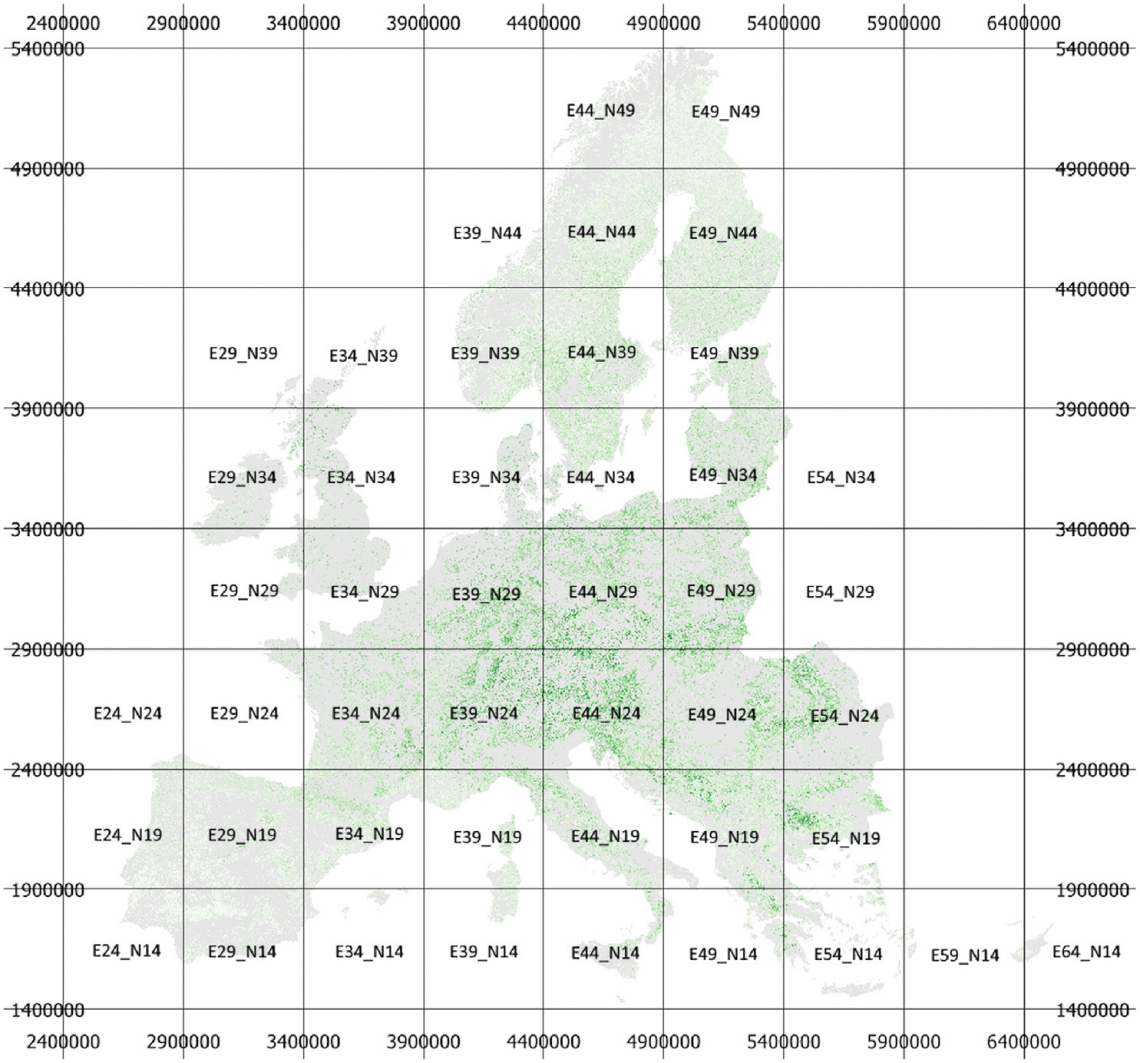
Maps are provided in 10 m spatial resolution in 500×500 km tiles in the EPSG:3035 - ETRS89-extended / LAEA Europe projection. Volume map as background in the image.

All maps are provided in 10 m spatial resolution in 500×500 km tiles in the EPSG:3035 - ETRS89-extended / LAEA Europe projection (Fig. 2). The file naming follows the following pattern:

‘Year’_’variable’_’tile’.tif, with variable names as defined in Table 1.

For example, the standard deviation map of volume for the 500×500 km grid tile with the lower left corner E 3 900 000 m and N 2 400 000 m is ‘2020_stdev_vol_E39_N24.tif’.

The maps fully cover a total of 40 European countries. In addition to the 27 EU countries, this includes Albania, Andorra, Bosnia and Herzegovina, Holy See, Liechtenstein, Monaco, Montenegro, North Macedonia, Norway, San Marino, Serbia, Switzerland and United Kingdom.

**Fig. 3:**
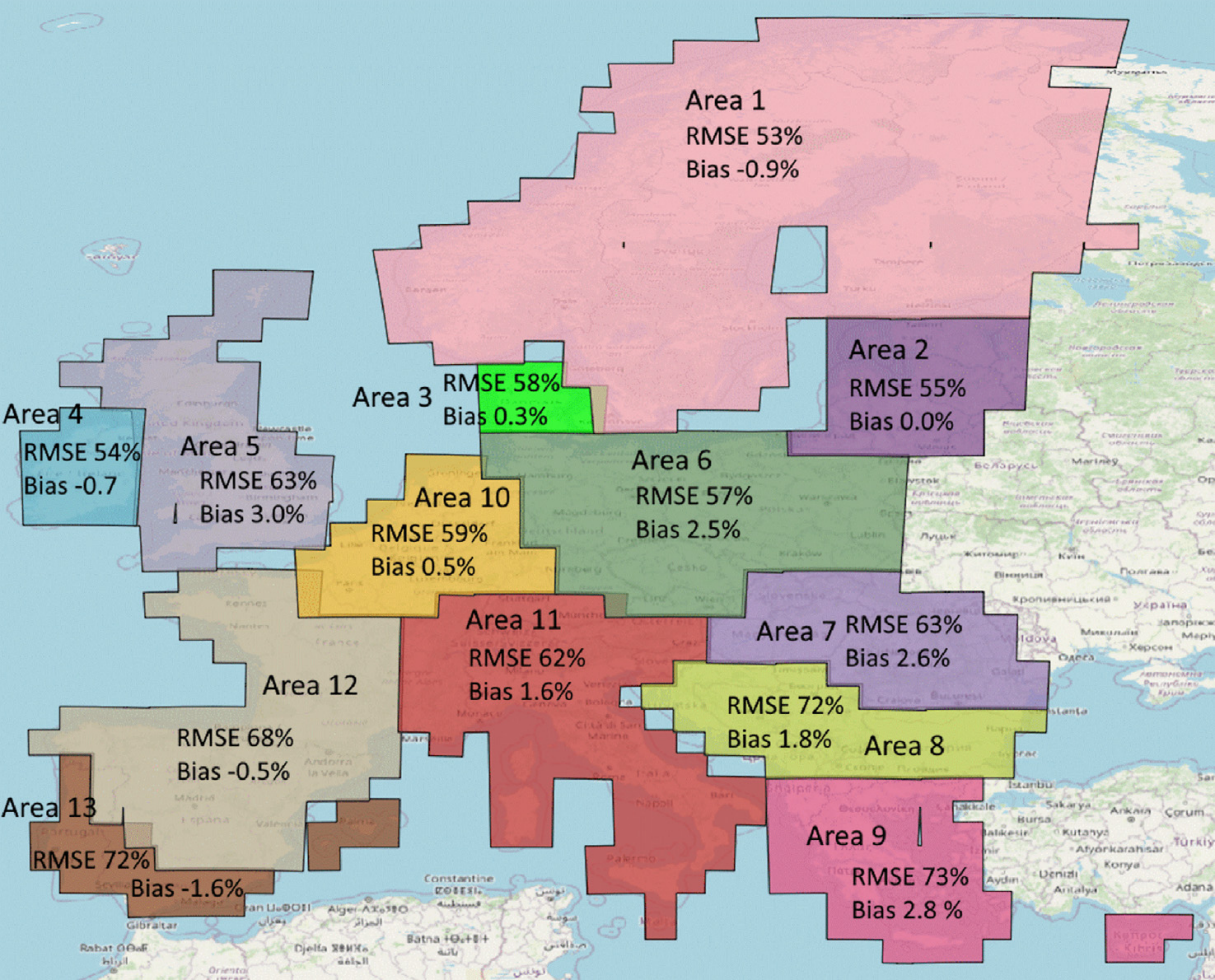
Relative plot level accuracies of AGB by processing areas.

The maps were produced using 13 separate processing areas (see the next section for more details). Error metrics for each of the processing areas are provided in Table 2 and Fig. 3.

**Table 2:** Plot level error metrics for the 13 processing areas calculated from the validation plots. Areas where plots were available and the plot location was used in the selection of the neighbours are in **bold**, whereas areas where plots were not available are in *italic*.

Processing area	Vol (m^3^/ha)					AGB (t/ha)					Con% (%)				
	RMSE	RMSE%	Bias	Bias%	R^2^	RMSE	RMSE%	Bias	Bias%	R^2^	RMSE	RMSE%	Bias	Bias%	R^2^
**Area 1**	75.7	58.2	-0.8	-0.6	0.54	40.2	53.2	-0.7	-0.9	0.54	22.3	30.4	1.1	1.5	0.52
*Area 2*	124.5	57.8	0.3	0.1	0.38	70.0	55.4	0.0	0.0	0.39	22.8	33.7	0.6	0.9	0.61
*Area 3*	187.7	55.4	0.7	0.1	0.05	103.4	58.0	0.5	0.3	0.08	22.6	37.7	-0.2	-0.3	0.73
**Area 4**	129.8	64.8	-0.5	-0.3	0.53	77.8	54.0	-1.0	-0.7	0.48	28.4	42.1	0.1	0.1	0.58
*Area 5*	146.4	72.0	9.3	4.6	0.38	90.6	63.4	4.3	3.0	0.31	27.0	50.6	0.7	1.4	0.67
**Area 6**	177.4	56.6	8.2	2.6	0.29	107.5	56.6	4.8	2.5	0.24	22.3	36.8	0.3	0.5	0.72
*Area 7*	201.0	61.9	7.9	2.4	0.27	127.2	63.4	5.3	2.6	0.15	24.8	41.3	-0.4	-0.7	0.65
*Area 8*	181.3	73.6	6.0	2.4	0.37	110.4	72.0	2.7	1.8	0.25	24.9	59.1	-0.1	-0.2	0.69
*Area 9*	127.0	76.9	2.3	1.4	0.41	86.1	73.4	3.3	2.8	0.30	19.1	110.3	-0.9	-5.1	0.69
**Area 10**	168.6	61.7	1.7	0.6	0.30	98.1	59.5	0.8	0.5	0.23	24.1	71.6	0.1	0.3	0.68
**Area 11**	178.2	64.3	5.2	1.9	0.31	103.6	62.3	2.7	1.6	0.24	24.2	61.8	0.1	0.3	0.69
**Area 12**	115.0	74.6	-0.9	-0.6	0.48	74.4	67.6	-0.6	-0.5	0.41	25.2	64.8	-0.3	-0.8	0.69
*Area 13*	49.5	83.9	-2.1	-3.5	0.60	44.6	71.6	-1.0	-1.6	0.47	26.5	65.5	-0.5	-1.2	0.68

Error metrics have been calculated by using the validation plots. For the processing areas where NFI plots are available, this approach can be expected to produce reliable error metrics. However, for the processing areas that do not have plots (i.e. Areas 2, 3, 5, 7, 8, 9 and 13), the error metrics should be treated with caution. For these areas, the mapping and validation plots were manually compiled using plots from ecologically similar regions. Although the selection was made with best available knowledge of the areas, the error metrics for the areas without field plots may be overly optimistic.

**Fig. 4:**
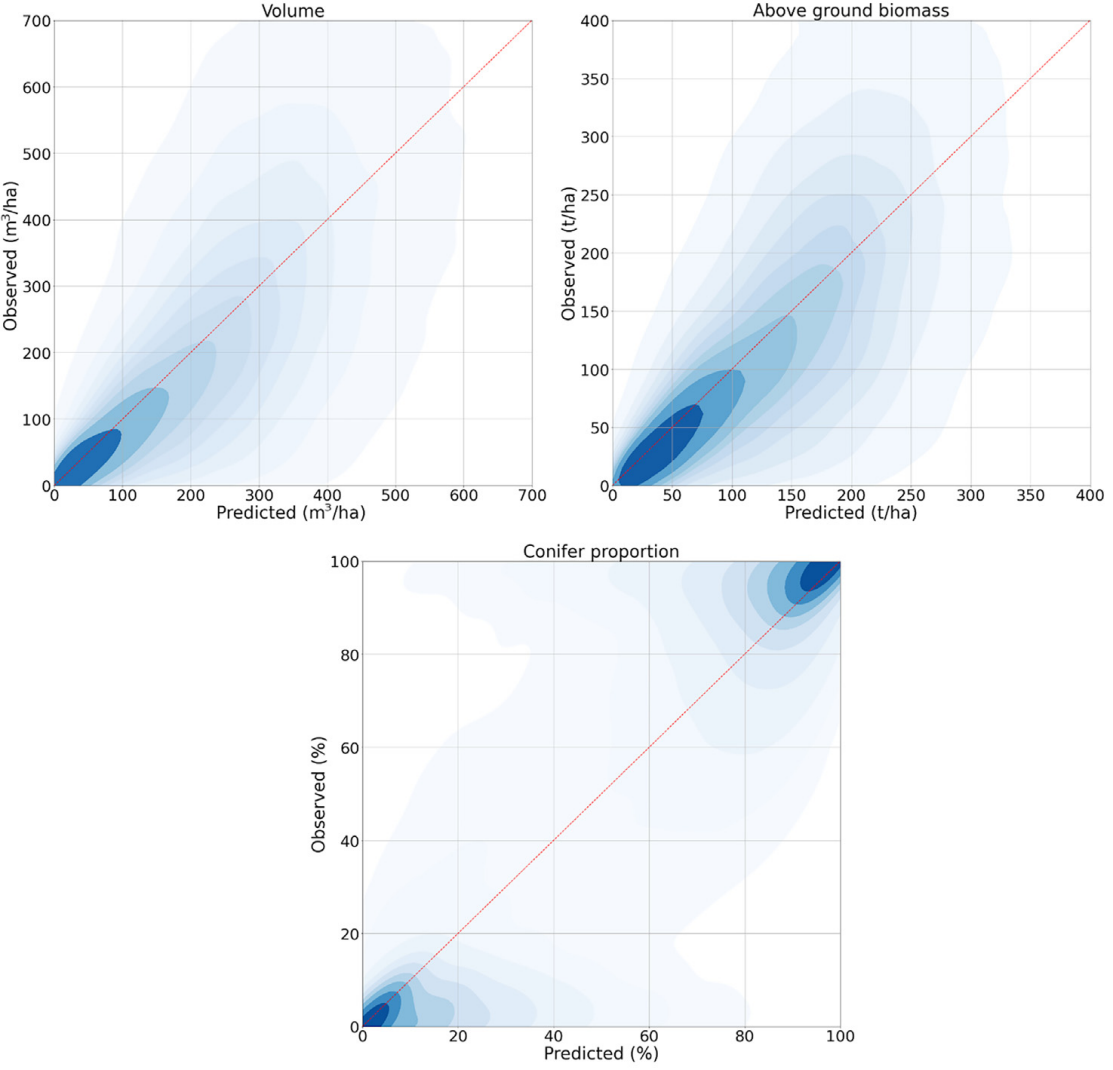
Scatterplots of observed and predicted Vol, AGB, and DCP based on the validation data. Darker colors indicate a higher density of observations.

Scatterplots based on the validation data show that the bulk of the data follow the 1:1 line. However, high values tend to be underestimated and low values tend to be overestimated (Fig. 4). These tendencies are evident in the change of bias for different size classes of forest. While the maps are on average nearly unbiased on European level (1.4 t/ha or 1.0 % of the 137 t/ha mean AGB), there is a clear overestimation for small biomass classes and increasing underestimation with increasing biomass (Table 3).

**Table 3:** Bias of AGB values for different biomass classes.

AGB bin (t/ha)	0-124	125-249	250-374	375-499	500->
**Bias (t/ha)**	38.3	-3.5	-87.1	-174.0	-351.1
**Bias%**	66.3	-1.9	-29.1	-42.6	-58.7

## 4 Experimental Design, Materials and Methods

### 4.1 NFI Field Data Processing

**Fig. 5:**
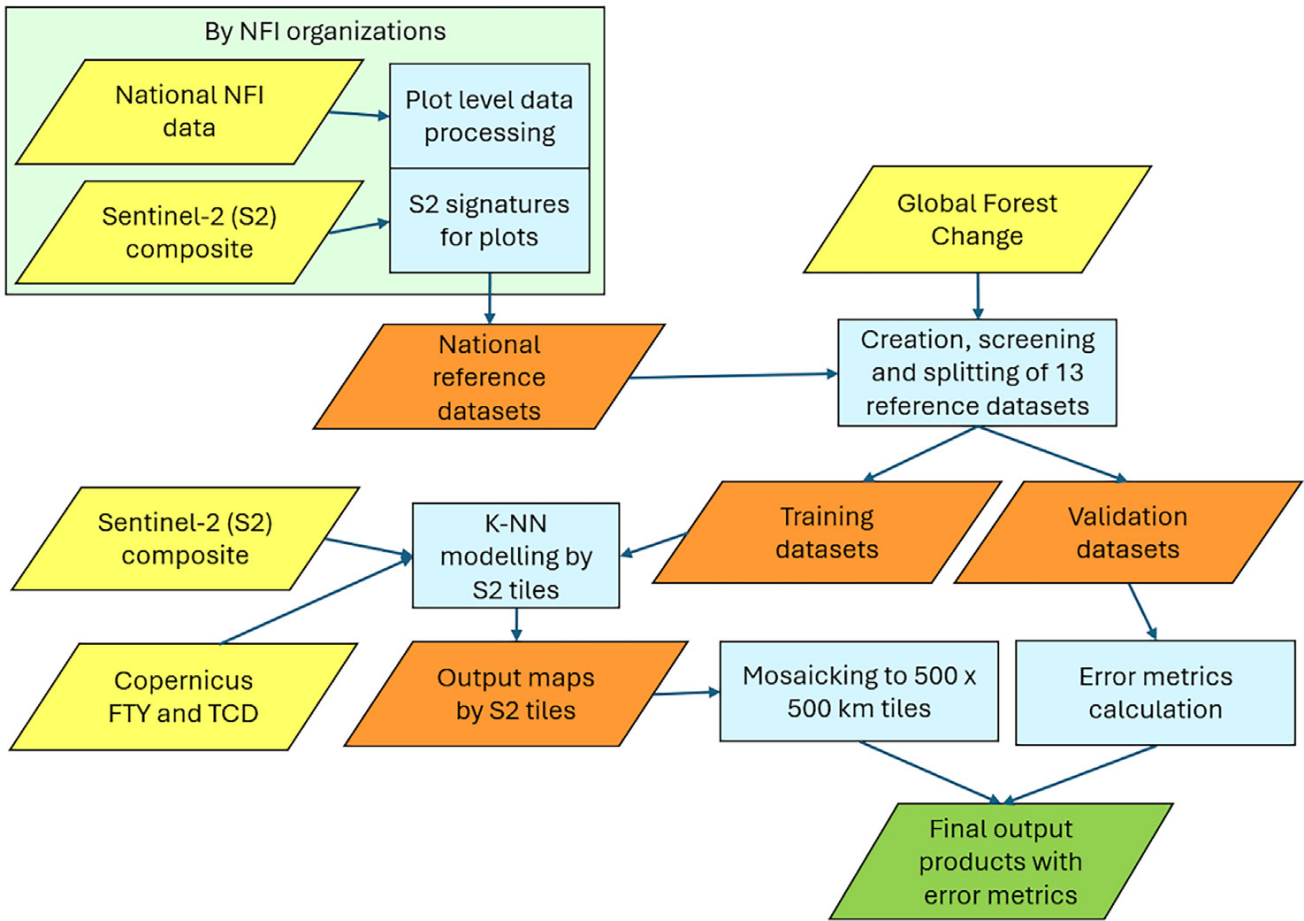
Workflow chart of data processing. Original data sources highlighted in yellow, intermediate in orange and the final output data in green. Further details of the datasets and processing steps can be found in the text.

The workflow chart of the data processing is illustrated in Fig. 5. The plot level data calculations of harmonized forest structure variables and the extraction of Sentinel-2 spectral signatures for plots were conducted by national NFI organizations. Detailed definitions of the harmonized volume (V) and above-ground biomass (AGB) can be found at [8] (variable ids 2 and 4). The deciduous-coniferous proportion (DCP) was calculated on plot level as the proportion of AGB of coniferous species [9] at the plot. The deciduous proportion is the inverse of the DCP value.

**Fig. 6:**
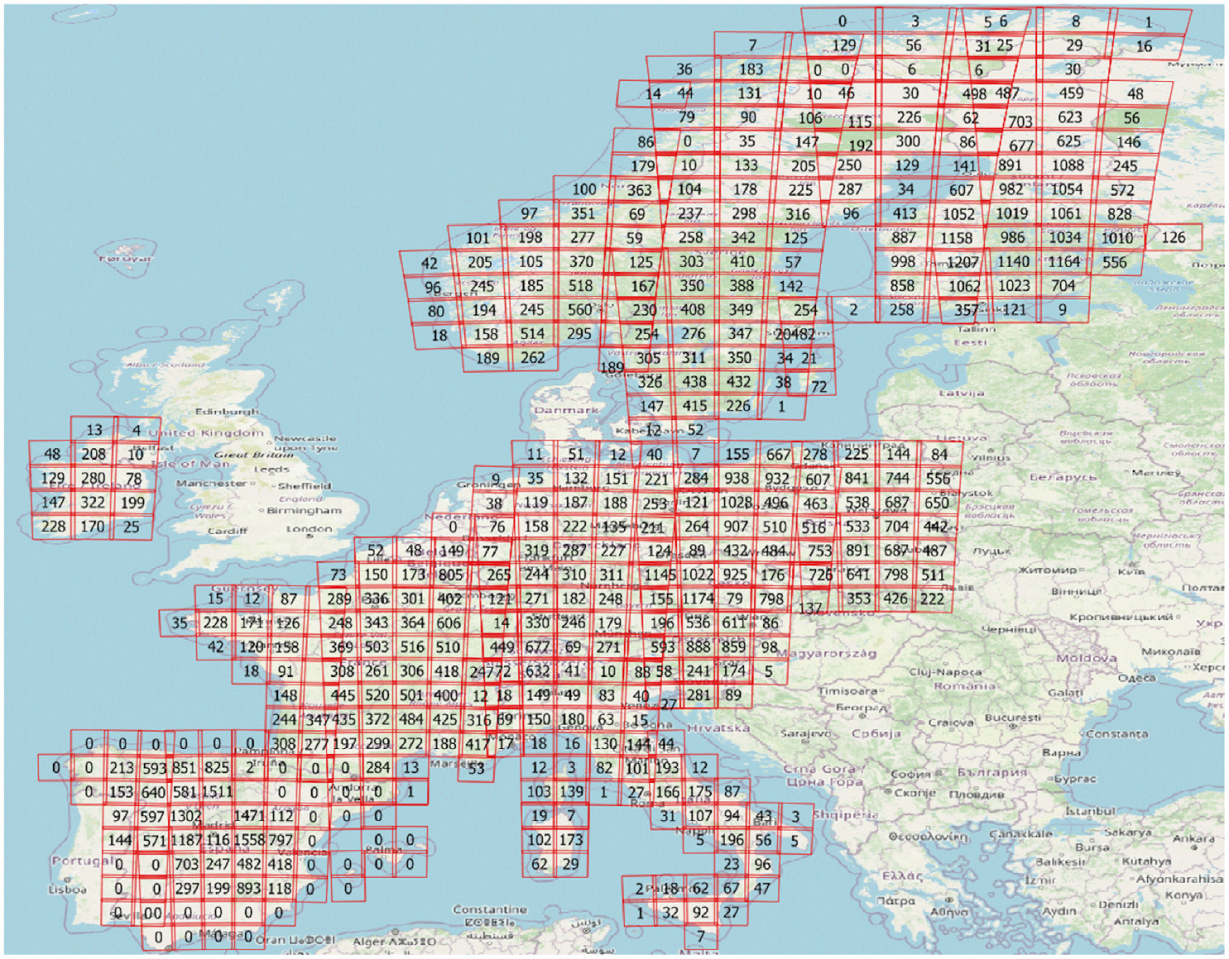
Number of field plots within Sentinel-2 tiles in countries that provided NFI data.

As a rule, plots measured 2019-2021 were used, to minimize the temporal difference between the field measurement and the Sentinel-2 observation (2020). However, in some countries the temporal range was extended either to include a sufficient number of plots or to include measurements from geographic areas that otherwise would not have been covered (Table 4). Because the Sentinel-2 and Copernicus data are organized in overlapping tiles, some sample plots were used more than once for modelling. The spatial distribution of the plots is shown in Fig. 6. All European NFIs used in the creation of the maps use representative probability sampling designs to select field sample plots that are used to create official statistics of forest resources. More detailed description of the NFI designs can be found e.g. in [10,11].

**Table 4:** Overview of the NFI data used in the mapping.

Country code	Measurement years	Number of plots used for mapping	Number of plots used for validation	Plots/forest area (N/km^2^)
AT	2019-2021	3816	1908	0.14
BE	2019-2021	934	466	0.29
CH	2019-2022	2168	1083	0.26
CZ	2018-2020	6902	3450	0.36
DE	2017	5735	2867	0.08
ES	2018-2021	12592	6296	0.10
FI	2019-2021	21432	10716	0.16
FR	2019-2021	12390	6195	0.11
IE	2020-2022	1185	592	0.24
IT	2018-2019	2813	1406	0.05
NO	2019-2021	5908	2954	0.07
PL	2019-2021	15754	7877	0.26
SE	2019-2021	8602	4300	0.06
SI	2018	566	283	0.07
TOTAL	-	**100797**	**50393**

### 4.2 Remote Sensing and Auxiliary Data Processing

The following remotely sensed data were used in the production of the maps (Fig. 5):

• A European-wide Sentinel-2 mosaic of composite images for the year 2020 [12] was used as the primary remote sensing data source. The image compositing algorithm using Level 2A surface reflectance products is described in Miettinen et al. [13]. The final composite images included seven spectral bands (B2, B3, B4, B5, B8, B11 and B12), all resampled into 10 m spatial resolution.
• Copernicus High Resolution Layers for forest in 10 m resolution with reference year 2018. We used Forest Type (FTY; coniferous forest, broadleaved forest, non-forest) and Tree Cover Density (TCD; 0-100 %) [14].
• Global Forest Change (GFC) 30 m resolution forest canopy loss product [15]. We used version 1.9 that included the years of forest canopy losses between and including 2001 and 2021.

The Sentinel-2 and Copernicus products created the feature space when choosing the nearest neighbours in the k Nearest Neighbour mapping (see more details below). The Global Forest Change product was used only in screening plots that had experienced changes after the field measurements.

The Sentinel-2 bands and the TCD data had continuous values and the weighted mean for a circle with a size of 100 m^2^ centred on each NFI sample plot was calculated from them. The pixel proportions covering the circle were used as weights. For the categorical variables (FTY and GFC), the weighted mode was calculated for the same circle. The weighted mode was the category with the greatest sum of weights. The calculations were done using R by each NFI organization, in order to use the exact plot coordinates. The standardized R scripts for this task are available as a git repository [16].

### 4.3 Application of the kNN Approach

The k Nearest Neighbour (kNN) approach was used with training data including forest target variables (V, AGB, and DCP) and the corresponding remotely sensed variables (seven Sentinel-2 bands, TCD, and FTY) for each NFI plot. Furthermore, 1 km INSPIRE grid cell locations (northing and easting) were included in the feature space to enable utilization of the plot location when selecting the nearest neighbours. The kNN model was run with the same parameters (k=7, Euclidian distance, weighing neighbours inverse to distance) in all processing areas. All features used in the modelling had equal weights.

The data were screened to (1) exclude cloud contaminated observations (based on composite image quality band accepting only values greater than 4000) and (2) exclude plots where changes had been detected by GFC since 2018 (or a year before the earliest plot measurements). The screened data were sorted by volume and every third plot was assigned to the validation data set. The remaining two thirds of the plots were used for the mapping (Table 4). The same dataset was used to predict all the three variables simultaneously in a multivariate manner.

**Fig. 7:**
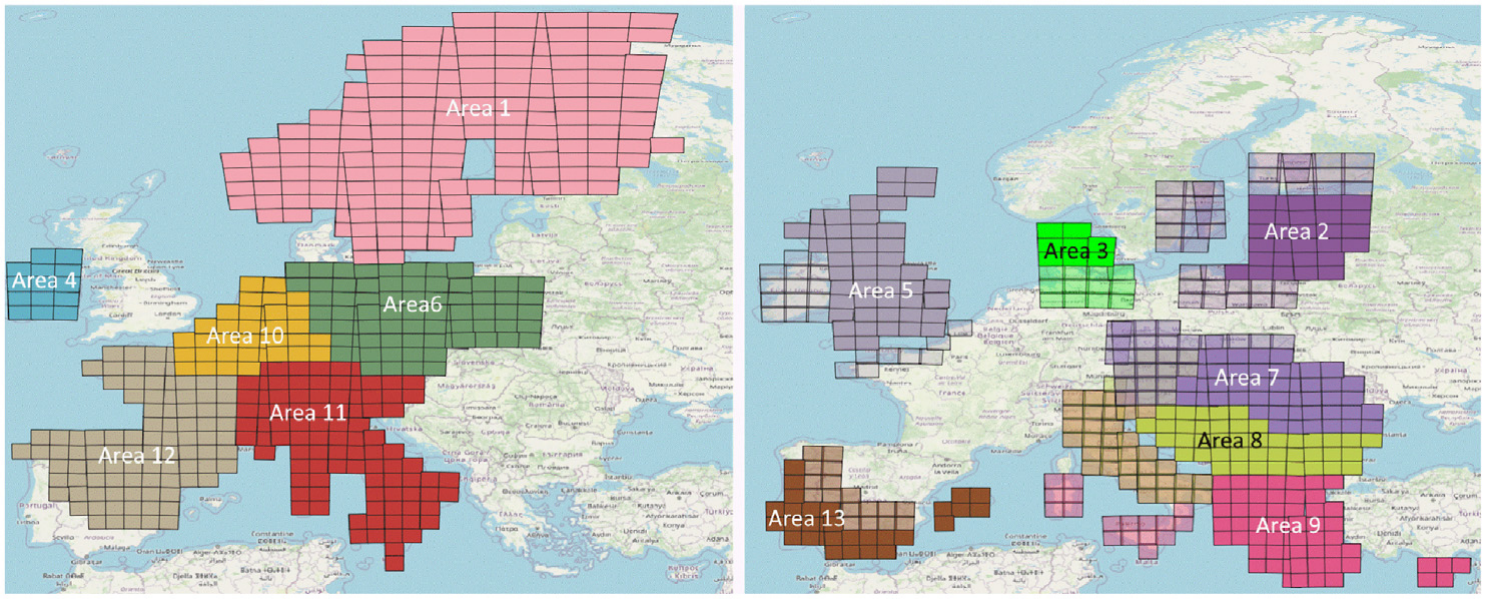
Sentinel-2 tiles colorized by processing area. Left: processing areas where NFI plots were available and location information was used in modelling. Right: processing areas where NFI plots were not available and location information was not used in modelling (except for northing in Area 2). Plot sourcing areas are shown in transparent color.

For the production of the maps, 13 processing areas were created taking into account the geographical areas of Europe and the availability of field sample plots (Fig. 7). Six of the processing areas contained NFI plots, while the remaining processing areas did not have any NFI plots. For those areas without NFI plots, training data were created using plots from ecologically similar sourcing areas. Mean Vol ranged between 130 and 326 m^3^/ha in the processing areas, whereas mean AGB values ranged between 76 and 202 t/ha. The proportion of conifer-dominated plots ranged from more than 70 % to less than 40 %.

In regions with NFI plots, the processing area borders were located in the middle of countries, but all plots from the countries that were (even partially) covered by a processing area were used for mapping. This approach ensured that in adjacent processing areas plots from both sides of the processing area border were used, resulting in a smooth transition between the processing areas with no visible changes in the map attributes at the borders of the processing areas.

For areas where NFI plots were available, the INSPIRE 1 km grid northing and easting were used as features in the selection of the nearest neighbours. This approach allowed utilization of large training datasets while still ensuring that the plots used for mapping were from ecologically similar areas. For countries where NFI plots were not available, training data were constructed with nearest available NFI plots from neighbouring countries. In these cases, the INSPIRE 1 km grid locations were not used among the attributes to search for the closest neighbours. An exception was processing area 2 (Fig. 7), where northing was used.

All processing was conducted in the Forestry TEP [17] by Sentinel-2 tiles. A total of 745 Sentinel-2 tiles were processed.

Euclidean distance was used to select the nearest neighbours in the feature space. The k-NN prediction ${\hat{y}}_{p}$ for pixel p is given by

(1) $${\hat{y}}_{p}=\;\sum_{l}w_{l}y_{l}$$

where y_l_ is the vector of observations and w_l_ is the weight of the l’th nearest neighbor with l=1.….k. Weights inverse to the Euclidean distance were used. In addition to the prediction, we calculated the standard deviation ${\hat{s}}_{p}$ of the nearest neighbors for each pixel as a measure of uncertainty

(2) $${\hat{s}}_{p}=\;\sqrt{\frac{\sum_{l}{\left(y_{l}-\;{\hat{y}}_{p}\right)}^{2}}{k}}$$

Root-mean-square-error (RMSE), bias and R^2^ were used to evaluate the mapping results. These metrics were calculated by predicting the response variables for the validation plots.

(3) $$RMSE=\;\sqrt{\frac{\sum_{i}{\left(y_{i}-\;\hat{y_{i}}\right)}^{2}}{n}}$$

(4) $$Bias=\;\frac{\sum_{i}\left(y_{i}-\;\hat{y_{i}}\right)}{n}$$

(5) $$R^{2}=1-\frac{\sum_{i}{\left(y_{i}-\;\hat{y_{i}}\right)}^{2}}{\sum_{i}{\left(y_{i}-\;\bar{y_{i}}\right)}^{2}}$$

where *y_i_* represents the reference values, *ŷ_i_* represents the predicted values, i=1.….*n* indexes the observations, and *n* is the number of observations in the validation database. The RMSE and bias values were also compared to the mean value of the variable in the validation plots, deriving relative metrics, denoted as RMSE% and Bias%.

The above error metrics were calculated by using the validation plots from the same processing areas, where available. To investigate the potential effects of utilizing data gathered from other geographical areas to derive error metrics, predictions were made for Czechia and Poland (which are both included in processing Area 6), without using the NFI data from the country in question. The results were validated with the validation data from the country.

The error metrics were calculated using (1) Area 6 data, and (2) Area 6 data excluding the mapping plots from the respective country (Table 4). In both cases. validation plots only from the respective country were used. In case 2), location information was not used in the model, making it comparable to those processing areas that do not have NFI plots available.

It can clearly be seen that in both cases the predictions with the national NFI plots produce clearly lower relative RMSE values (Table 5). This highlights the importance of the availability of local reference data when using empirical methods such as the k-NN approach. The biases, on the other hand, behave more erratically and vary strongly between the training dataset and can be high in some cases. Overall, the target variable predictions for the processing areas without NFI plots need to be treated with caution. There is an increased probability of high biases caused by the use of NFI plots from other geographical areas.

**Table 5:** Error metrics with and without NFI plots for two example countries.

Country	Training dataset	Vol		AGB		Con%	
		RMSE%	Bias%	RMSE%	Bias%	RMSE%	Bias%
**Czechia (CZ)**	**Current map**	53.8	2.3	56.9	-0.6	35.7	-0.3
	**No CZ plots**	63.2	1.2	66.0	-15.7	39.8	0.6
							
**Poland (PL)**	**Current map**	54.6	3.1	53.1	3.4	35.4	0.7
	**No PL plots**	65.8	22.1	65.6	23.1	39.1	4.4

## Limitations

It is important to note the underestimation of high and overestimation of low volume and biomass values in the maps (Fig. 4, Table 3). It is therefore not recommended to use the maps as a sole means for estimation of forest statistics for a given interest area. The maps are primarily meant to be used in model-assisted estimation where model-related biases can be mitigated, and field-based estimates improved.

In addition to the uncertainty of the accuracy of the maps in areas that have no field plots (discussed in the previous section), another issue potentially affecting the consistency of the map is the difference in the NFI designs. That means that, e.g. the temporal and spatial frequency of reference plots differ between the countries. In some countries, the plots used for the creation of the maps were measured a few years before the satellite data acquisition, while in most countries the temporal match is very good (Table 4). The effects of these differences could not be evaluated for the current set of maps but should be a topic for further investigation.

## Ethics Statement

The authors confirm that they have read and follow the ethical requirements for publication in Data in Brief and confirm that the current work does not involve human subjects, animal experiments, or any data collected from social media platforms.

## CRediT authorship contribution statement

**Jukka Miettinen:** Conceptualization, Methodology, Formal analysis, Writing – original draft, Writing – review & editing. **Johannes Breidenbach:** Conceptualization, Methodology, Writing – original draft, Writing – review & editing, Funding acquisition, Project administration. **Patricia Adame:** Resources, Data curation. **Radim Adolt:** Resources, Data curation. **Iciar Alberdi:** Resources, Data curation. **Oleg Antropov:** Methodology, Software. **Ólafur Arnarsson:** Resources, Data curation. **Rasmus Astrup:** Project administration, Writing – review & editing. **Ambros Berger:** Methodology, Resources, Data curation, Writing – review & editing. **Jón Bogason:** Resources, Data curation, Writing – review & editing. **Gherardo Chirici:** Resources, Data curation. **Piermaria Corona:** Resources, Data curation. **Giovanni D'Amico:** Resources, Data curation, Writing – review & editing. **Jiří Fejfar:** Resources, Data curation. **Christoph Fischer:** Resources, Data curation. **Florence Gohon:** Resources, Data curation. **Thomas Gschwantner:** Resources, Data curation, Writing – review & editing. **Johannes Hertzler:** Resources, Data curation. **Zsofia Koma:** Project administration, Writing – review & editing. **Kari T. Korhonen:** Resources, Data curation. **Luka Krajnc:** Resources, Data curation. **Nicolas Latte:** Resources, Data curation. **Philippe Lejeune:** Resources, Data curation. **Andrew McCullagh:** Resources, Data curation. **Marcin Mionskowski:** Resources, Data curation. **Daniel Moreno-Fernández:** Resources, Data curation, Writing – review & editing. **Mari Myllymäki:** Methodology, Data curation, Writing – review & editing. **Mats Nilsson:** Resources, Data curation. **Jérôme Perin:** Resources, Data curation. **Juho Pitkänen:** Resources, Data curation, Writing – review & editing. **John Redmond:** Resources, Data curation. **Thomas Riedel:** Resources, Data curation, Writing – review & editing. **Johannes Schumacher:** Resources, Data curation. **Lauri Seitsonen:** Software, Visualization. **Laura Sirro:** Methodology, Formal analysis. **Mitja Skudnik:** Resources, Data curation. **Arnór Snorrason:** Resources, Data curation. **Radosław Sroga:** Resources, Data curation. **Berthold Traub:** Resources, Data curation. **Björn Traustason:** Resources, Data curation. **Bertil Westerlund:** Resources, Data curation. **Stephanie Wurpillot:** Resources, Data curation.

## Data Availability

ZenodoHigh-Resolution Pan-European Forest Structure Maps: An Integration of Earth Observation and National Forest Inventory Data (Original data).
